# Characterization of the *Plasmodium* Interspersed Repeats (PIR) proteins of *Plasmodium chabaudi* indicates functional diversity

**DOI:** 10.1038/srep23449

**Published:** 2016-03-21

**Authors:** Xue Yan Yam, Thibaut Brugat, Anthony Siau, Jennifer Lawton, Daniel S. Wong, Abdirahman Farah, Jing Shun Twang, Xiaohong Gao, Jean Langhorne, Peter R. Preiser

**Affiliations:** 1 School of Biological Sciences, Nanyang Technological University, 637551, Singapore; 2 Francis Crick Institute, Mill Hill Laboratory, London, UK; 3 Karolinska Institutet, Stockholm, Sweden

## Abstract

*Plasmodium* multigene families play a central role in the pathogenesis of malaria. The *Plasmodium interspersed repeat* (*pir*) genes comprise the largest multigene family in many *Plasmodium spp.* However their function(s) remains unknown. Using the rodent model of malaria, *Plasmodium chabaudi*, we show that individual CIR proteins have differential localizations within infected red cell (iRBC), suggesting different functional roles in a blood-stage infection. Some CIRs appear to be located on the surface of iRBC and merozoites and are therefore well placed to interact with host molecules. In line with this hypothesis, we show for the first time that a subset of recombinant CIRs bind mouse RBCs suggesting a role for CIR in rosette formation and/or invasion. Together, our results unravel differences in subcellular localization and ability to bind mouse erythrocytes between the members of the *cir* family, which strongly suggest different functional roles in a blood-stage infection.

Multigene families are present within sub-telomeric and telomeric regions of most chromosomes of malaria parasite species. The best-known example is the *var* gene family of *Plasmodium falciparum* that is expressed on the surface of infected red cells (iRBC), undergoes mutually exclusive expression and enables the parasite to evade host immunity by transcriptional switches (a process called antigenic variation)^1^. *Var* gene products also mediate binding to host cells such as endothelial cells (cytoadherence) or uninfected red blood cells (rosetting), a property associated with virulence^2^,^3^. This gene family is not found in other *Plasmodium* species infecting humans, rodents or simians. By contrast, the *pir* (*Plasmodium* interspersed repeat) multigene family is present in all *Plasmodium* genomes so far sequenced. This large family includes *rif* (~200) and *stevor* (~35) in *P. falciparum*, and *cir* (~200), *bir* (~180), *yir* (~800), *kir* (~68), *cyir* (~256), *vir* (~350) in *P. chabaudi*, *P. berghei*, *P. yoelli*, *P. knowlesi*, *P. cynomolgi*, and *P. vivax* respectively^4^,^5^,^6^. Despite their discovery more than a decade ago, the function of *pir* genes is not yet understood. Their number and variant nature, and their potential location on or near the surface of infected red blood cells (iRBC) would support the idea that PIR may be important for antigenic variation and immune evasion. In addition, recent *in vitro* studies have shown that VIR protein can mediate cytoadherence to endothelial cells^7^,^8^, and STEVORs and RIFINs participate in red blood cell binding during the formation of rosettes and/or erythrocyte invasion^9^,^10^,^11^.

While *in vitro* studies on *rifs*, *stevors* and *virs* have revealed important information about location and potential function, without suitable experimental models for *P. falciparum* and *P. vivax* it is not possible to validate their importance *in vivo*. To determine the importance of *pir*, and whether different PIR perform different functions, it is necessary to be able to study them in a tractable experimental model that will allow *in vivo* and *in vitro* studies. A rodent malaria parasite, such as *P. chabaudi*, is a robust model for studying the interaction of host cells and immune responses with PIR. This parasite gives rise to an acute parasitemia followed by a prolonged chronic infection^12^,^13^. It is known to express serologically distinct surface antigens on the iRBC^13^,^14^,^15^, thus allowing a study of the relationship between antigenic variation and antibody responses. Furthermore *P. chabaudi* iRBCs accumulate or sequester in organs during a blood-stage infection^12^,^16^,^17^,^18^, and *P. chabaudi* iRBCs have been shown to bind to uninfected RBC and form rosettes^19^. However the antigens involved in the immune evasion or the cytoadhesion of iRBCs to host cells have yet to be determined.

Using RNA sequencing and microarray studies, we, and others previously showed that the CIR multigene family can be classified into distinct subfamilies (A and B), and that there are differences in the level and timing of transcription between *cir* members during the blood stages of a *P. chabaudi* infection^17^,^20^. Recently, Otto *et al.* has provided a more comprehensive classification of the *pir* genes using new highly accurate and almost complete reference genomes of rodent parasites where the pirs were classified into ‘long’ (L) and ‘short’ (S) form^21^. Strikingly, despite the use of different tools and methods of classification, it appears all the members of the previously classified *cir* subfamily A belong to the newly classified L type and all the members of the *cir* subfamily B belong to the S type. In this study, we have used several approaches to locate CIR proteins in infected RBC including IFA (indirect immunofluorescence assay) with a panel of anti-CIR antibodies, and live cell imaging of fluorescently tagged CIR. These experiments show differential subcellular localization of CIRs during the asexual blood stages, which is affected to some extent by their L and S classification, strongly indicating different roles between members of the *cir* multigene family. Importantly, we demonstrate that some CIR proteins are located on the edges of merozoites, and a subset of them binds to host RBC suggesting a possible role in RBC invasion and/or rosetting. Together, these data suggest that individual *cir* members can play different functional roles during the blood stages of the infection.

## Results

### CIR proteins have different subcellular locations in *P. chabaudi*-infected red blood cells

To determine the subcellular localization of CIRs in the asexual blood stage parasites, we first generated antibodies against four *cir* genes expressed as Histidine (His)-tagged recombinant proteins (PCHAS_000730, PCHAS_140090, PCHAS_110020 from subfamily S and PCHAS_000950 from subfamily L; Figs S1–3), which were previously shown to exhibit different transcriptional peaks throughout the intra-erythrocytic developmental cycle^20^. Sequence alignments of the above four His-tagged CIR proteins with two other recombinant CIR proteins (PCHAS_00100 and PCHAS_040110) showed a certain degree of similarity and the presence of a highly conserved motif (Q/E)YAILW(F/L) in the region against which polyclonal antibodies were raised (Fig. S2).

Western blot analysis showed that each of the four antisera at 1:64 000 dilution were specific to their respective His-tagged CIR proteins (Fig. S3A), however, two of the antisera (anti-950 and anti-140090) still showed some cross reactivity to other CIR protein (PCHAS_00730) at this high dilution. Using a pool of the four CIR antisera at 1:16 000 dilution (with each individual serum at 1:64,000 dilution in the pool) showed stronger reactivity to their individual CIR protein, and also faintly detected the other two CIR recombinant proteins (PCHAS_00100 and PCHAS_040110) (Fig. S3A). This is not surprising as the sequence alignment data indicated stretches of amino acid similarity and a highly conserved peptide motif within the region the CIR antibodies were raised against (Fig. S2).

**Figure 1:**
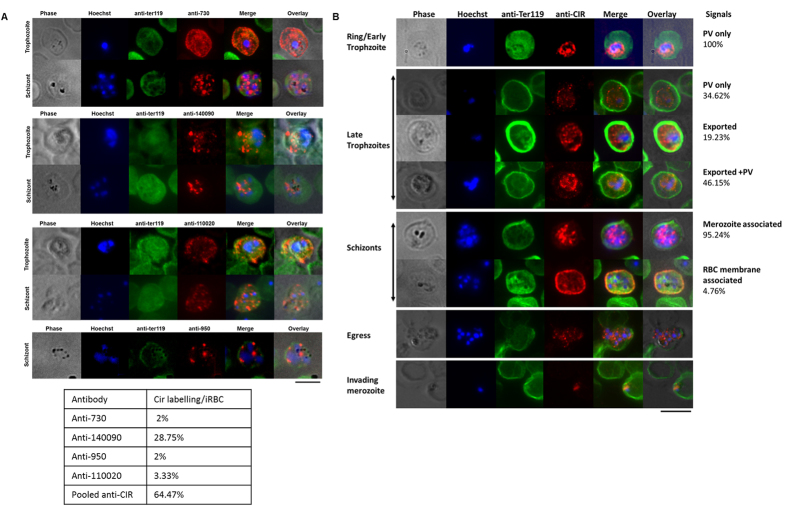
CIR sub-cellular localization during the asexual blood stages of *P. chabaudi.* Representative pictures of immunofluorescence assay on acetone/methanol fixed red blood cells (RBC) infected with *P. chabaudi* AS. Parasites nuclei were labeled with Hoechst 33342 (blue) and the mouse erythrocyte membranes with an anti-Ter119 antibody (green). (**A)** CIR proteins were labeled with single CIR-specific polyclonal antibodies (red). Table show the percentage of infected red cells positively labeled with respective antibodies used (50–100 cells were counted per experiment). Scale bars represent 5 μm. (**B)** CIR proteins were labeled with a pool of polyclonal anti-CIR antibodies (red). The phase contrast image is shown in grey. The percentage of infected red cells showing CIR localization is shown on the right hand side (20–26 cells counted per experiment). Scale bar represent 5 μm. PV: inside the parasitophorous vacuole, Exported: inside the red blood cell cytoplasm (see also Figs S1A, S2, S3A and S4).

Immunofluoresence assays (IFA) using the four antisera individually (Fig. 1A) showed that all four CIRs were closely associated with individual merozoites as dots during the schizont stage of parasite development. However, in the trophozoite stage, all CIRs were exported to the cytoplasm of iRBC with PCHAS_00730 being more closely associated with the RBC membrane while no signal was detected for PCHAS_00950 at this stage. Quantification of individual CIR differential localization at trophozoite stage was not possible as individual antisera detected only few cells expressing the respective CIRs, suggesting that individual parasites express different members of CIRs. Therefore the four antisera were pooled to maximise the proportion of iRBCs staining positively for CIR with approximately 64.5% of iRBC staining positively with the pooled anti-CIR Abs, in contrast to the individual antisera shown in the table in Fig. 1A and Fig. S4.

Using the pooled anti-CIR antisera, different subcellular localizations of CIRs were observed (Fig. 1B). All CIRs were found to be associated with the parasitophorous vacuole (PV) during the ring/early trophozoite stages. As the parasites matured to late trophozoites, CIRs remained within the PV in approximately 35% of iRBC, were exported to the host cell cytosol in 19% of iRBC, or were present in both the PV and host cell cytosol in approximately 46% of iRBC. During the schizont stage of parasite development, CIRs were closely associated with individual merozoites in approximately 95% of iRBC and were associated with the iRBC membrane in approximately 5% of iRBC. In the few cases where free merozoites were detected it appeared that upon schizont rupture and egress, CIRs were associated with the invading merozoite.

**Figure 2:**
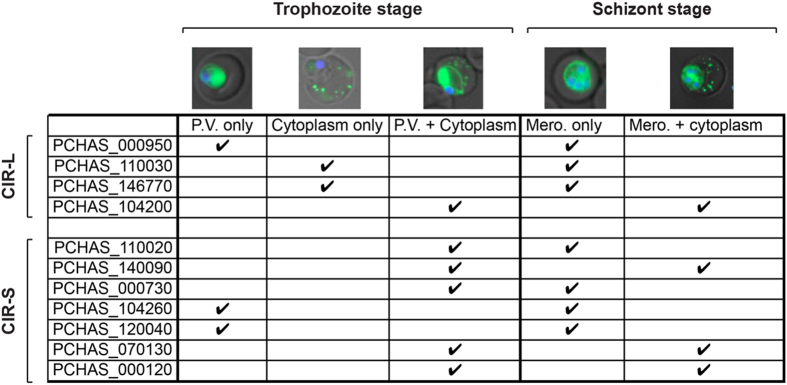
CIR proteins have differential localization. Table summarising results obtained by live cell imaging of transgenic *Plasmodium chabaudi* parasites expressing CIR proteins tagged with GFP (green). Representative pictures of the trophozoite and schizont stages of each CIR member are shown (upper panel). The subfamilies and gene accession number of the each CIR member are indicated along the left hand side of the table. Parasites nuclei were labeled with Hoechst 33342 (blue). The phase contrast image is shown in grey. (See also Fig. S5). PV: inside the parasitophorous vacuole, Mero.: associated with merozoites.

As IFA was performed using the pooled anti-CIR antisera, it was not possible to determine whether individual CIR localized to different sites within the infected RBC. To investigate this further, we generated transgenic lines of *P. chabaudi* expressing 11 GFP-tagged CIRs under the control of the constitutive promoter *ef1a*, including the four CIRs described above and a range of CIR proteins belonging to different subfamilies, previously shown to be highly transcribed *in vivo*^20^. Live cell imaging of the trophozoite and schizont stages of the individual transgenic CIR-GFP parasites (Fig. 2 and Fig. S5) showed that CIRs were exported to the host cell cytosol, whilst others remained within the PV and were associated with merozoites. Overall, 3 out of 4 members (~75%) of the CIR-L subfamily members and 5 out of 7 members (~71%) of the CIR-S subfamily were exported to the RBC cytosol during the trophozoite stage of the parasite cycle. Furthermore, CIR proteins from both subfamilies were associated with the individual merozoites during the schizont stage (Fig. 2 and Fig. S5).

When comparing the *cir* transcriptional data from Lawton *et al.*^20^ (Fig. S6A) with the localization data of CIR using the individual CIR-specific antibodies generated in this study (Fig. S6B), we observed that, although some *cirs* are expressed during the ring and trophozoite stages, proteins could still be detected during the schizont stage, indicating that the protein is stable during parasite maturation. On the other hand no CIR is detected during the early ring stage indicating that there is no or very limited transfer of the protein from the merozoite to newly invader red blood cells. However, we observed no correlation between the timing of transcription of *cir* genes and the localization of CIR proteins. Furthermore, when CIR proteins were constitutively expressed after episomal transfection, similar localization was observed between the transfected and the wild type protein (Fig. S6B,C). Therefore the timing of *cir* gene expression does not seem to have an impact of the location of CIR proteins.

**Figure 3:**
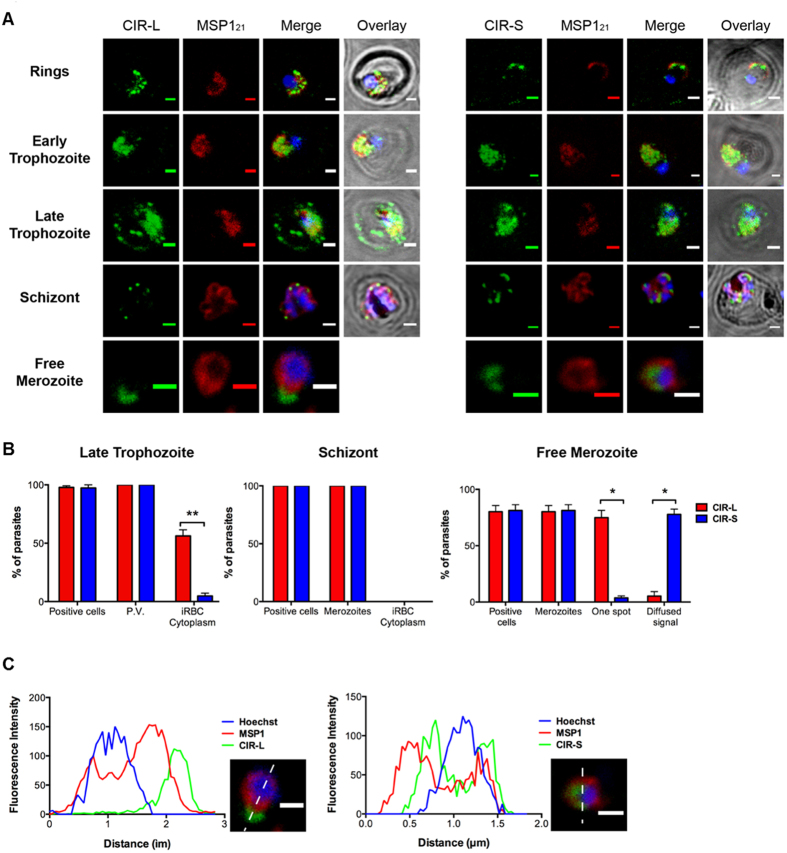
CIR-L and -S proteins have different localization during the trophozoite and merozoite stages. (**A)** Representative pictures of immunofluorescence assay on acetone/methanol fixed red blood cells (RBC) infected with *P. chabaudi* AS. Anti-peptides antibodies, specific for CIR-L or -S proteins, were used (green) (see also Figs S1B,C and S3B,C). The membrane of the parasitophorous vacuole and the edges of the merozoites were stained with an anti-MSP1_21_ antibody (red) and parasites nuclei were labeled with Hoechst 33342 (blue). Brightfield is shown in grey. Scale bars represent 1 μm. **(B)** During the late trophozoite, schizont and merozoite stages, the percentage of parasites for which CIR-L or -S have been located inside the infected RBC (iRBC; positive cells), the parasitophorous vacuole (P.V.), around segmented merozoites, or the iRBC cytoplasm were determined. For the merozoites, signals seen in a single spot or in a more diffuse pattern were counted. Bars represent the mean ± S.E.M in at least 3 independent experiments (10–100 cells counted per experiment). (Mann-Whitney; *P < 0.05; **P < 0.01; ***P < 0.001). **(C)** Fluorescence intensity of Hoechst 33342 (blue), MSP1_21_ (red) and CIR-L or -S (green) along the white dotted line drawn across a free merozoite (bottom right panels). Scale bars represent 1 μm.

As only a small number of proteins could be studied by transfection (11 out of 200 *cir*s found in the genome), antibodies against conserved amino-acid motifs present in a majority of the members from each subfamily were generated (Figs S1B,C and S3B,C) to determine whether any difference could be observed in cellular localization between CIR L and S. The conserved amino-acid sequences recognized by our anti-peptide antibodies are located in the C-terminal region of CIRs. This region is highly hydrophobic and was not included in the recombinant CIR proteins to maximize their expression (Fig. S1). It was therefore not possible to test the specificity of our anti-peptide antibodies directly on recombinant CIR L or CIR S proteins. However, both anti-sera detected their respective CIR-subfamily specific peptide in ELISA assays (Fig. S3B). Although some CIR-L proteins are longer than CIR-S proteins this is not true for all members of this clade (CIR-L have molecular weights between 33 kDa and 168 kDa while CIR-S are between 23 kDa and 66 kDa). In fact, 50% of CIR-L proteins and 99% of CIR-S proteins have a molecular weight between 33 kDa and 66 kDa. Accordingly, when used in western blot experiments against parasite lysates, both anti-CIR-L and anti-CIR-S antisera recognized bands between ~30 kDa and ~70 kDa (Fig. S3C). Different patterns were observed between the two antisera and these bands were not recognized by the pre-immune serum and absent in uninfected RBC lysates. Overall these results clearly indicate that our anti-peptide antibodies specifically recognize the CIR-specific amino-acid sequences. Immunofluorescence assays using these anti-CIR-L/CIR-S peptide antibodies showed that in ring and early-trophozoite stage parasites both CIR L and S appear to be localized within the parasite cytoplasm. It is interesting to note that CIR staining appears to be concentrated in distinct spots in the ring parasites rather than the more diffuse cytoplasmic location seen in the early-trophozoite stages (Fig. 3A). A clear difference in localization between subfamily L and S is seen during the late-trophozoite stage, when CIR L proteins were observed beyond the PVM in distinct spots in the RBC cytosol (Fig. 3A). Quantification of the export pattern of CIR L and S in late trophozoite stage parasites showed that approximately 60% of the iRBCs, which stained positively for CIR-L proteins demonstrated export into the RBC cytosol, compared with only approximately 10% of the iRBCs stained with CIR-S antibodies (Fig. 3B). At the schizont stage, CIR L and S proteins were no longer present in the RBC cytoplasm but were associated with the segmenting merozoites (Fig. 3A). Confocal microscopy analysis of free merozoites showed different localization of CIR-L and -S proteins (Fig. 3B,C). CIR-L proteins were observed in a single focus at the tip of the merozoite as indicated by the shift between the signal obtained with anti-MSP1_21_ and anti-CIR-L antibodies (Fig. 3C). This pattern was observed in approximately 80% of merozoites with anti-CIR-L antibody, but only 5% of merozoites with anti-CIR-S. By contrast, a more diffuse signal around the nucleus, internal to that of MSP1_21,_ was observed with the anti-CIR-S antibody (in approximately 80% of cells compared to approximately 2% of cells with anti-CIR-L) (Fig. 3B). Taken together these data would indicate that while the CIR-L/CIR-S distinction may not be sufficient to definitively predict their sub-cellular localization during the asexual blood stages, there is a clear tendency for CIR L proteins to be exported into the red cell cytoplasm and to the tip of the merozoite, while CIR S appear to be preferentially retained within the parasite.

Of importance, IFA with these anti-peptide antisera showed that 80–100% of the parasites were positively stained with both anti-CIR-L and anti-CIR-S antibodies from the late trophozoite to the merozoite stage (Fig. 3B), indicating that each parasite expresses at least 2 *cir* genes (one from each subfamily).

### CIR binds to host red cells with different efficiency

Although they can have different localization during the intra-erythrocytic life cycle of the parasite, every CIR protein tested was associated with merozoites. We, therefore, speculated that one function of CIR may be to bind RBC. We employed 3 different assays to assess this possibility.

**Figure 4:**
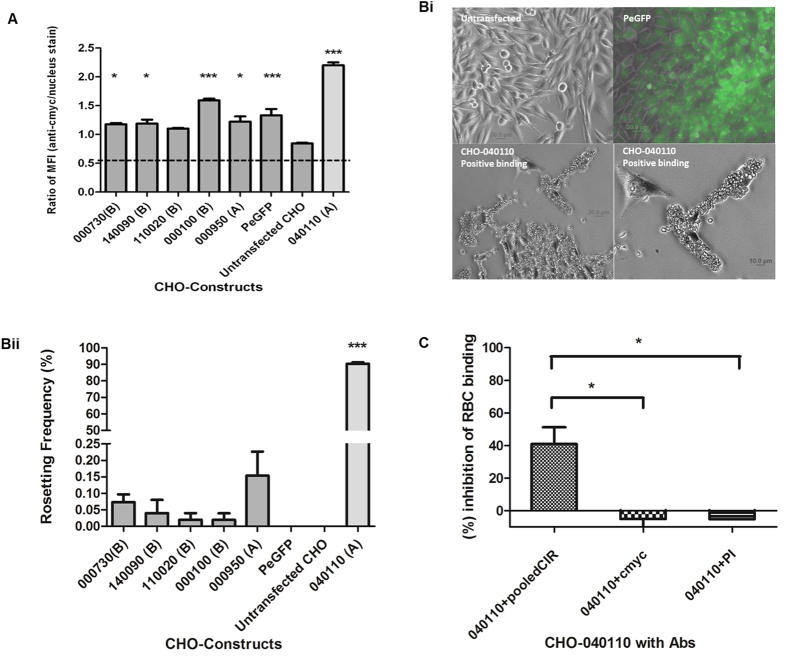
CIR proteins mediate binding of mouse erythrocytes to CHO cells *in vitro.* (**A)** Ratio of Mean fluorescence intensity (MFI) of anti-C-myc antibodies/nucleus stain on transfected CHO cells expressing CIR proteins (see also Fig. S1A and S2) and eGFP and the untransfected CHO cells. Bars represent the mean ± S.E.M. of three independent experiments. **(Bi)** Representative pictures of mouse red blood cells (RBC) binding to CHO cells expressing CIR on their surface. Untransfected and surface expressing eGFP CHO cells were used as negative controls. Scale bars are shown in the respective pictures. **(Bii)** Rosetting frequency represents the percentage of CHO cells binding to at least 5 mouse RBCs. Bars represent the mean ± S.E.M. of three independent experiments (~3000 CHO cells were counted per experiment). **C)** Erythrocyte binding assay were performed with CHO cells expressing PCHAS_040110 on their surface in the presence of pooled polyclonal anti-CIR antibodies, anti-C-myc, or preimmune (PI) sera. (See also Fig. S3A and D). The percentage of inhibition was calculated according to the rosetting frequency of CHO cells expressing PCHAS_040110 in absence of antibodies. Bars represent the mean ± S.E.M. of three independent experiments. (~1000 CHO cells were counted per experiment). All statistical analysis performed with One way ANOVA with post hoc (Bonferroni); *P < 0.05; **P < 0.01; ***P < 0.001.

In the first approach, we expressed six CIR proteins lacking the transmembrane domain and the conserved C-terminus (Figs S1A and S2) on the surface of CHO cells. After selection on the neomycin resistance marker, surface expression of the transfected proteins on the transfected cells was validated by performing IFA with anti-C-myc antibody against the C-myc tag located at the C-terminus of the cloned protein and the signal was quantified using a fluorescence plate reader (Fig. 4A). The ability of these cells to bind uninfected RBC was determined (Fig. 4B). In three independent experiments, CHO lines transfected with all six CIRs tested bound to red blood cells, albeit with different efficiency. Five of the transfected lines showed weak binding less than 0.2% of the cells bound to 5 or more RBC. By contrast, 90% of cells transfected with PCHAS_040110 had 5 or more RBC bound (Fig. 4Bii), indicating that some CIR proteins are able to mediate strong binding to mouse RBC while others do not. No binding was observed for either untransfected CHO cells or CHO cells transfected with the control plasmid PeGFP expressing GFP protein.

To assess whether the binding observed was specific to CIR we tested the CIR antisera generated in this study for their ability to disrupt binding of uninfected RBC to CHO cells transfected with the strongly binding CIR PCHAS_040110. As our pooled CIR antibodies had detected the recombinant PCHAS_040110 in western blot (Fig. S3A) it was also important to establish whether PCHAS_040110 exposed epitopes were recognized by the pooled anti-sera when expressed on the surface of CHO cells. IFA using pooled CIR sera and anti-C-myc antibodies on CHO transfected cells expressing PCHAS_040110 confirmed that the pooled anti-CIR antibodies do recognize the surface expressed PCHAS_040110 protein (Fig. S3D). No inhibition of RBC binding was observed with either the C-myc antibody or pre-immune (PI) sera while the pooled anti-CIR antibodies reduced binding by approximately 40% (Fig. 4C). These results indicate that CIR antibodies can disrupt the binding activity of PCHAS_040110 to the mouse RBC in line with a specific binding interaction between CIR and RBC.

**Figure 5:**
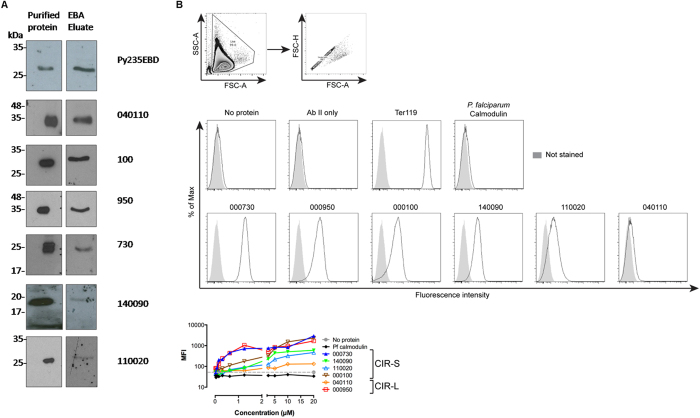
CIR proteins bind to red blood cells *in vitro.* Mouse red blood cells were incubated with recombinant histidine (His)-tagged proteins (see also Fig. S1A and S2). **(A)** 2.5 μg of soluble proteins were used. Cells were washed and erythrocyte-bound proteins were then eluted and analysed by western blot. *Plasmodium yoelii* 235EBD was used as positive control. The presence of the recombinant protein before (purified protein) and after incubation with mouse RBC (EBA Eluate) was detected by a monoclonal anti-His antibody. **(B)** Binding of recombinant proteins to mouse RBC was, this time, analysed by flow cytometry using an anti-His tag primary antibody and an Alexa 488 anti-mouse IgG secondary antibody. As controls, cells were either not stained, incubated with His-tagged *Plasmodium falciparum* calmodulin, antibodies only (no protein), secondary antibody only (Ab II only) or an anti-Ter119 antibody (recognising the surface of all RBC) was used as primary antibody. Live single cells were selected (upper panels). The histograms show the fluorescence intensity of red blood cells incubated with 20 μM of recombinant proteins (middle panels). Mean fluorescence intensity (MFI) according to the concentration of recombinant protein used is shown in the lower panel. Each dot represents the mean of 2 independent experiments.

Soluble recombinant His-tagged CIR proteins (Figs S1A and S2) were assessed for their ability to bind uninfected RBC using two additional assays. In the first, CIR proteins were incubated with mouse RBC. Unbound proteins were separated by centrifugation of the cultures through oil, bound proteins were then eluted from the RBC and detected by anti-His Tag antibody on Western blots^22^. PCHAS_040110, PCHAS_000950, PCHAS_000100, PCHAS_000730 and the positive control protein Py235 proteins were clearly detected on western blot (Fig. 5A). By contrast, CIR proteins: PCHAS_140090 and PCHAS_110020 were faintly detected only after longer exposure time, again indicating heterogeneity in the efficacy of CIR binding to RBC.

His-tagged recombinant CIR proteins were also assessed for binding to RBC by flow cytometry (Fig. 5B). In agreement with the RBC Binding Assay shown in Fig. 5A, RBCs incubated with recombinant PCHAS_000730, PCHAS_000100, PCHAS_140090, PCHAS_110020, and PCHAS_000950 all showed a significant increase in mean fluorescence intensity (MFI, Fig. 5B) compared with cells incubated without protein, with the detecting antibody only, or with a His-tagged control protein (*Plasmodium falciparum* calmodulin) confirming that the long N-terminal region of CIR proteins can bind to RBC. For each recombinant CIR, the mean fluorescence intensity increased with the concentration of recombinant protein used. However, above 5 μM, the MFI reached a plateau indicating that the binding of CIR proteins to RBCs could be saturated.

All three RBC binding assays are in general agreement and show that these expressed CIRs can bind to RBC, albeit with different efficiencies. CHO cells transfected with PCHAS_040110 exhibited strong binding to RBC as well as in the RBC-binding assay but showed almost no binding in the flow cytometric assay. By contrast, recombinant proteins PCHAS_000730, PCHAS_000100, PCHAS_140090, PCHAS_110020, and PCHAS_000950 showed clear binding in both the RBC-binding assay and the flow cytometry assay, while little RBC binding was detected when these proteins were expressed in CHO cells. Differences in conformation of CIR expressed on CHO cells or as recombinant soluble proteins may explain this discrepancy, and highlight the importance of using multiple approaches when studying RBC binding of parasite proteins. However, recombinant PCHAS_000100, PCHAS_000950 and PCHAS_000730 showed a stronger binding than PCHAS_140090 and PCHAS_110020 in both mouse RBC and flow cytometry-based binding assays (as shown by a higher MFI, and an increase of MFI at low concentrations of recombinant protein) indicating that, in addition to their differences of expression and sub-cellular localization, CIR proteins also have different capacities to bind red blood cells. Taken together these three different assays show for the first time that a subset of CIR proteins can bind to the host RBC indicating a potential role of CIRs in rosetting or RBC invasion.

### Binding of CIR expressed on CHO cells to mouse RBC is mediated by a protein-protein interaction

**Figure 6:**
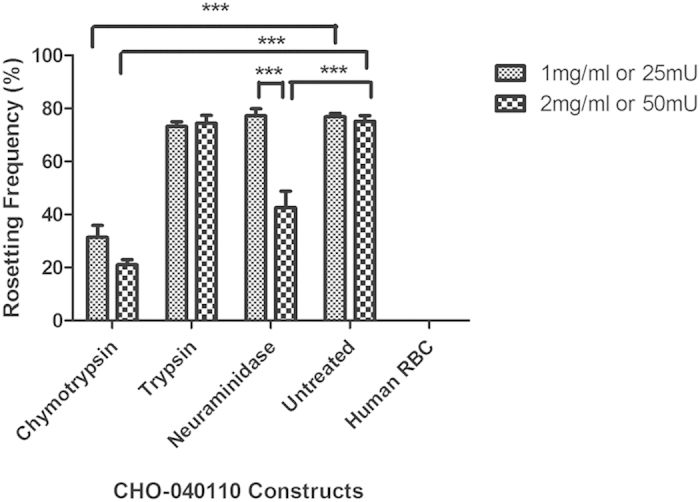
CIR binding to red blood cells is mediated by protein-protein interaction. Erythrocyte binding assay were performed with CHO-PCHAS_040110 line with different concentrations of enzyme and antibody treated and untreated mouse erythrocytes and human RBC. Rosetting frequency represents the percentage of CHO cells binding to at least 5 RBCs. Bars represent the mean ± S.E.M. of three independent experiments (~1000 CHO cells were counted per experiment). All statistical analysis performed with One way ANOVA with post hoc (Bonferroni); *P < 0.05; **P < 0.01; ***P < 0.001.

To determine whether CIR binding to RBC is the result of a protein-protein interaction, we investigated whether binding was sensitive to enzyme treatment of the RBC. Mouse RBCs were incubated with different concentration of the proteases chymotrypsin, and trypsin or neuraminidase prior to incubation with CHO cells expressing PCHAS_040110 (selected because of strong RBC binding described in Fig. 4). The binding of this transfected CHO cells to mouse RBC was sensitive to chymotrypsin, as the rosetting frequency dropped significantly to approximately 35% and 20% with chymotrypsin at 1 mg/ml and 2 mg/ml respectively, compared with the cells incubated with untreated RBC (Fig. 6). Binding to RBC was also sensitive to neuraminidase but only at a high concentration of 50 mU. The rosetting frequency at 50 mU of Neuraminidase was significantly reduced to approximately 50% of that observed with 25 mU of Neuraminidase. The binding to RBC was insensitive to trypsin. The RBC binding activity of CIR is specific to mouse RBC only as the CHO cells expressing PCHAS_040110 did not bind to human RBC (Fig. 6).

Together, our data show that the binding of CIRs to RBCs can be saturated (Fig. 5B), is chymotrypsin and dose-dependent neuraminidase sensitive, and specific to mouse RBC (Fig. 6), and thus strongly indicate that this process is mediated by a protein-protein interaction.

## Discussion

Despite the universal presence of the *pir* multigene family in the genomes of all *Plasmodium* species sequenced to date, its function(s) is not known. We show here that PIR proteins of *P. chabaudi* (CIR) are expressed during the entire asexual blood cycle but can have different subcellular localizations, partly influenced by their subfamily classification, strongly indicating different roles for these proteins in the erythrocytic life cycle. Furthermore we demonstrate that CIRs are located on the edges of merozoites and a subset of them can mediate binding to red blood cells via a protein-protein interaction, suggesting a role in red cell invasion. Together, these data strongly indicate that CIR proteins play different roles in the erythrocytic life cycle, some of which may be important for the survival of *Plasmodium* parasites.

CIRs are observed in all stages of the asexual life cycle; however their location changes over time. They are first detected within the parasitophorous vacuole (PV) during the ring stage, and then the RBC cytoplasm and on the edges of iRBC during the late trophozoite stage. During the schizont stage, some CIR proteins disappear from the red blood cell cytosol and are associated with the segmented merozoite before and upon egress. A previous study of *P. vivax* VIR proteins also indicated they could be found in several locations within the iRBC and close to the cell surface^7^. We observed close location of CIR with the cell surface in only a very small number of iRBCs, similar to a previous study of YIR of *P. yoelii*^7^,^23^,^24^. This suggests either not all PIR/CIRs are exported to the surface, or that the CIR proteins we selected for expression and generation of antibodies are not those predominantly exposed on the surface of red cells.

The different location of the members of the CIR family was particularly pronounced during the trophozoite stage of the parasite: some CIRs remained within the PV while others were exported to the RBC cytosol, or were present in both the PV and RBC cytosol. *Plasmodium chabaudi* episomally transfected with a plasmid ePL containing GFP-tagged *cir* under the control of *P. berghei ef1*α constitutive promoter^25^ showed that both CIR-L and CIR-S subfamily members were exported to the host-cell cytosol as dot-like structures during the trophozoite stage. Despite the limitations in expressing genes from episomal constructs using strong promoters and large fluorescent tags^26^, the export of CIR proteins in dot-like structures in the cytosol of iRBC and their presence around the merozoites agreed with the IFA with our anti-protein and anti-peptide antibodies, therefore confirming the results obtained by this technique. These dot-like structures in the RBC cytoplasm may be the same membranous structures as those recently described in *P. yoelii* and *P. berghei*^25^,^27^, which have been suggested to share similar functions to the Maurer’s clefts and act as a staging platform for protein sorting and trafficking to their final destinations^28^,^29^.

When comparing the data obtained from transcriptomic and immuno-fluorescence analyses, we observe no association between the timing of *cir* genes expression and the location of their product. It is therefore likely that the protein sequence, rather than the timing of expression, determine the final location of CIR proteins. While it was not possible to predict confidently the location of CIRs according to their allocation to the CIR-L or CIR-S subfamilies^20^ as shown by fluorescently-tagging individual CIRs, the anti-peptides antibodies, expected to recognize more members of each subfamily, indicated a greater tendency for CIR-L proteins to be exported out of the parasitophorous vacuole, and a differential distribution of CIR-L and CIR-S proteins on the surface of the merozoite. Strikingly, the different localization of CIR-L and -S proteins in the blood stages is reminiscent of the RIFINs of *P. falciparum* that is also divided into 2 subfamilies. As for the *cir*-L subfamily, the sequences of *rif*-A subfamily genes are also more variable than those in subfamily B, and RIFIN-A proteins have an insertion containing conserved cysteines, are more likely to be exported in the cytosol of the iRBC during the trophozoite stage and are associated with the apical end of the merozoites^20^,^30^. RIFIN-B proteins on the other hand tend to be located in the PV, and are found all around the merozoites. The similar cellular localization of CIR and RIFIN could reflect a conserved function between these multigene families during the blood stages of the infection. Our findings reported here therefore strongly support the idea, first suggested from a previous genomic analysis^5^, that the *pir* gene family is functionally related to the *rif* and *stevor* families of *P. falciparum*. Together these data provide strong evidence for different functions of different CIR/PIR proteins and demonstrate the relevance of rodent models to study their role *in vivo*.

Previous studies have shown the presence of surface antigens in *P. chabaudi* that differ between the primary peak of infection and the subsequent chronic infection^15^,^31^, however their identities remain unknown. The anti-CIR antibodies recognizing individual proteins stained only a few iRBC suggesting that, in a single infection, sub-populations of parasites may express different CIR proteins. Similar observations at the RNA level have been made for *yirs, virs*, and *rifs*^32^,^33^,^34^,^35^ with only a small number of genes transcribed in a single parasite. On the other hand, it appears that CIR-L and CIR-S proteins, as defined by anti-peptide antibodies, can coexist in the same iRBC, suggesting that more than one CIR protein is expressed in a single cell, in agreement with previous transcriptional analysis of *vir, yir* and *rif* transcripts^30^,^32^,^33^.

*Pir* gene expression is not as restricted as for the *var* gene family in *P. falciparum* where only a single gene is transcribed whereas all the others are silenced^1^. However, considering the very high number of genes present in the genome (838 *yirs*, 346 *virs*, 200 *cirs* and 200 *rifs*) the expression of *pir* genes still appears to be tightly controlled. The expression of only few CIRs in/on an individual iRBC, which are different between iRBC would be compatible with a role in antigenic variation and immune evasion, and here we show that recombinant CIR proteins are recognized by antibodies in plasma from mice that have recovered from a *P. chabaudi* blood-stage infection (Fig. S7), similar to the observations that antibodies to VIR are present after *P. vivax* infections^36^ and to RIFINs after *P. falciparum* infections^37^. The next step will be to determine which CIRs are on the RBC surface, whether the presence in the plasma of antibodies recognising these proteins is detrimental for the parasites and whether *cir* expression changes during the chronic phase of infection, allowing the parasite to escape this immune response.

The presence of some CIRs on the merozoite is also compatible with a role in binding to RBC during invasion. RIFINs and STEVORs have been implicated in the cytoadherence to uninfected RBC during the formation of rosettes^9^,^10^,^11^,^35^,^38^ and/or RBC invasion^10^. *P. vivax* iRBC can also bind to uninfected RBC in the absence of VARs^39^ indicating that other surface proteins, possibly VIR, are involved in that process. In line with this hypothesis, we show for the first time that a set of recombinant CIR proteins can bind to uninfected red cells and CIR expressed on the surface of CHO cells can form rosettes with mouse RBCs. Our results also demonstrate that CIR binding to RBCs, 1) can be saturated, 2) is chymotrypsin and dose-dependent neuraminidase sensitive and 3) is specific to mouse erythrocytes, strongly suggesting that this process is mediated by a protein-protein interaction. These results together with the presence of CIR on the edge of merozoite therefore suggest that these proteins could be involved in RBC invasion, a key mechanism of the *Plasmodium* life cycle. Furthermore, the CIR proteins bind RBC with a different efficiency, suggesting different affinities for the RBC receptor. This could influence their ability to invade new RBCs and would have an impact on parasite survival and virulence as described for the py235 multigene family^40^. Our results therefore highlight the importance of CIR during a malaria infection.

In summary, our findings demonstrate that CIR proteins have differential subcellular localizations in iRBC and merozoites, indicating different functions for different CIRs during a blood-stage infection with *P. chabaudi*. Recombinant CIRs can mediate binding to RBC suggesting their involvement in that mechanism implicated in parasite survival and virulence. These findings highlights the similarities with RIFINs of *P. falciparum* and suggest that the rodent malarias such as *P. chabaudi* could be potent models not just to understand the *vir* genes of *P. vivax*, but also to elucidate the role of the *rifs* of *P. falciparum*.

## Methods

### Ethics statement and animals

This study was performed in accordance with the NACLAR (National Advisory Committee for Laboratory Animal Research) guidelines under the Animal & Birds (Care and Use of Animals for Scientific Purposes) Rules of Singapore and the UK Animals (Scientific Procedures) Act 1986 (Home Office licence 80/2538). The protocol was approved by the Institutional Animal Care and Use Committee (IACUC) of Nanyang Technological University (NTU) of Singapore (Approval number: ARFSBS/NIE A002) and the National Institute for Medical Research Ethical Committee. All animals used in this study were obtained from Sembawang Laboratory Animal Center, National University of Singapore, or the specific pathogen free (SPF) unit at the MRC National Institute for Medical Research and subsequently housed under SPF conditions at NTU and MRC Animal Holding Unit.

### Recombinant proteins and Antibodies generation

To facilitate the expression of recombinant CIR proteins, a truncated fragment was synthesized in which the predicted transmembrane domain(s) and conserved C-terminus (based on the sequences available on http://www.genedb.org) were replaced by a 6-histidine tag (Figs S1 and S2).

Selected DNA sequence regions of the following cir genes: PCHAS_00730, PCHAS_140090, PCHAS_000950 and PCHAS_110020 were amplified by PCR from *P. chabaudi* genomic DNA (gDNA). They were cloned in frame into pET24a(+) vector (Novagen) and expressed in BL21-RIL strain (Stratagene, USA) as recombinant proteins. The fusion proteins were purified via Nickel column (Qiagen) according to manufacturer’s protocol. CIR proteins that tended to form aggregates in solution were further purified by gel elution before use for immunization of rats and generation of polyclonal CIR antibodies. 100 μg of purified proteins were injected subcutaneously into Wistar Rat with complete Freund’s adjuvant and subsequently 50 μg of proteins were injected at 2 weeks interval with incomplete Freund’s adjuvants. The rats were bled one week after the fourth immunization. Specificity of the rat polyclonal CIR antibodies was validated by immunoblotting using the recombinant proteins (Fig. S3A). Rat anti-730, anti-950, anti-110020 and anti-140090 antibodies (1:64,000 dilution); anti-pooled of the four CIR antibodies (1:16,000 dilution (each individual sera is at 1:64,000 in the pooled)); mouse monoclonal anti-His antibody (1:10,000 dilution) and rat pre-immune serum (PI) (1:100 dilution) were used. Secondary goat anti-rat HRP and goat anti-mouse HRP were used at 1:5000 for immunodetection (Fig. S3A). Soluble recombinant proteins were also further generated by optimizing the purification conditions and were used for subsequent assay.

Recombinant PCHAS_000100 and PCHAS_040110 were resynthesized (GENEWIZ) for efficient yeast expression. They were then expressed in *Pichia pastoris* using the PichiaPink^TM^ Expression system (Invitrogen) according to manufacturer’s instructions. Histidine-tagged MSP1_21_ was also expressed in *Pichia pastoris* using the pIC9 K vector^41^. These proteins were purified by binding on NiNTA agarose resin columns (Qiagen) and eluted with 200 mM imidazole. Proteins were then dialyzed against PBS using dialysis cassettes with a 10 kDa cut-off (Thermo-scientific) and stored at −80 °C before use.

Histidine-tagged *Plasmodium falciparum* Calmodulin was a kind gift from Judith Green and Anthony Holder (National Institute for Medical Research MRC, UK). Briefly the gene of interest was amplified from *P. falciparum* cDNA (made from parasites 36h post-invasion) and inserted into pET46Ek/LIC. Expression was then induced in BL21(DE3) cells and proteins were purified by hydrophobic interaction chromatography on a phenylsepharose column in presence of calcium. Purified proteins were dialyzed against PBS using dialysis cassettes with a 3 kDa cut-off (Thermo-scientific) and stored at −80 °C before use.

The mouse monoclonal NIMP23 antibody, recognizing *Plasmodium chabaudi* AS MSP1_21_, was expressed and purified as previously described^42^.

Peptides sequences KNMKKVINLAYGK (CIR-L specific) and QYLREKRKKAKRKVYNY (CIR-S specific) were derived from the conserved motifs identified by Lawton *et al.*^20^ in the *Plasmodium chabaudi* AS genome (Fig. S1). These peptides conjugated to KLH were used to immunize rabbits and generate anti-CIR-L and -S specific antisera (Harlan UK Hillcrest). Their specificity was tested by ELISA assays using the CIR-L and -S peptides conjugated to BSA, and immunoblot (1:1000 dilution for both antisera) on parasite and uninfected red blood cell lysates (Fig. S3B,C). For that purpose, and to avoid any future unspecific background due to a very high amount of haemoglobin, RBCs from infected and uninfected mice were first lysed with PBS, 0.15% saponin. Parasite fractions (containing the parasitophorous membrane and parasites) were then purified from infected RBCs after several centrifugations at 3000 rpm for 5 min to enrich the samples with parasite proteins. As a control, plasma membranes were purified from uninfected RBCs after several centrifugations at 20000 rpm for 30 min. Parasites fractions and uninfected RBCs membranes were then lysed for 10 min at RT with an equal volume of SDS buffer (50 mM Tris, 1% SDS, 5 mM EDTA, pH 8) and then boiled for 1 min. Protein extraction was completed by adding nine volumes of NP40 buffer (50 mM Tris, 150 mM NaCl, 0.5% NP40, 5 mM EDTA, pH8) and protease inhibitors. Lysates were then used in SDS PAGE gel electrophoresis. As loading controls, anti-Ter119 (Biolegend, RBC plasma membrane protein) and anti-Aldolase (Agrisera, soluble protein) antibodies were used.

### Plasmid construction

Full length coding sequences of *cir* genes were generated by chemical gene synthesis (GeneScript) and were amplified by PCR for cloning into plasmid ePL vector with GFP tag under a constitutive promoter (*P. berghei ef1a*) (in house made vector^25^,) for transfection of *P. chabaudi.* For the transfection of CHO cells, the same region of the N-terminus of CIRs used to generate recombinant proteins was amplified and cloned into the commercially available pDisplay vector (Invitrogen).

### *Plasmodium chabaudi* parasites preparation and transfection

*Plasmodium chabaudi chabaudi* (AS) parasite line from^20^ was used in this study. 6–8 weeks old males BALB/c or females C57BL/6 mice were infected with cryopreserved stocks of *P. chabaudi* by intraperitoneal injections and parasitemia was monitored by Giemsa-stained blood smears. When parasitemia reached 30–40%, the mice were scarified and parasites were harvested as previously described^43^. Briefly, trophozoites and schizonts stages were separated using 50–60% Histodenze (Sigma) gradient. Schizonts were cultured at 37 °C *in vitro* in RPMI1640 containing 20% FBS (Gibco) till maturity. Mature *P. chabaudi* schizonts were transfected with the ePL plasmid containing the gene of interest using the Basic Parasite Nucleofector solution kit II (Lonza) with Amaxa electroporator according to published protocol^43^,^44^,^45^. Transfected parasites were then injected intravenously into BALB/c mice and selected under pyrimethamine drug (Sigma). Transfectants appear usually between 14–21 days.

### Immunofluorescence assay (IFA) and Live cell imaging

Since *P. chabaudi* has a synchronous erythrocytic life cycle, blood could be collected at several time points to study the different asexual stages. Thin smears were prepared and fixed in cold acetone:methanol (9:1) for 10 min at 4 °C and air-dried. Slides were then incubated with blocking buffer (3% BSA in PBS) for 10 min at RT and subsequently incubated with primary antibodies for 1 hr at 37 °C or overnight at 4 °C. Anti-CIR, anti-peptides and anti-MSP1_21_ antibodies were used at 1:100, 1:10 and 1:50 dilution respectively (in PBS, 3% BSA). After three washes with PBS, slides were incubated 1 hr at 37 °C with secondary antibodies [Alexa 594 goat anti-rat secondary antibody (1:350 dilution; Biolegend), Alexa 488 goat anti-rabbit IgG or Alexa 594 anti-mouse antibodies (1:200 dilution; Sigma)] and/or with mouse anti-Ter 119 (1:500 dilution) conjugated with Alexa 488 to stain mouse red cells membrane and washed again. Nuclei were stained with Hoechst 33342 (1 μM in PBS). Slides were then mounted with vectashield medium (Vector laboratories) and viewed under Olympus IX71 fluorescence microscope using 100X oil immersion objective equipped with Olympus DP30BW camera. Image acquisition was also performed using a Leica SPE confocal microscope, using 63x/1.30 NA oil objectives. Image treatment and analysis were performed using Olympus, Leica and ImageJ 1.43u (NIH) software.

For live cell imaging of transgenic parasite with GFP tagged CIRs, the parasites were incubated 10 min with Hoechst 33342 (1 μM in RPMI1640) for nuclei staining. The cells were then viewed immediately between slide and coverslip as described above. Images were processed using Olympus DP manager version 2,2,1,195 and ImageJ 1.44o.

### CHO mammalian cell line transfections and selection of transfectants

CHO cells were cultured in F12 medium (Hyclone) supplemented with 10% FBS (Gibco) and Pen-Strep antibiotics (Hyclone) at 37 °C in 5% CO_2_. Commercial pDisplay plasmid with C-myc tag at the C-terminal of the cloned gene of interest was transfected into CHO mammalian cells using Lipofectamine LTX & Plus reagent (Invitrogen) in 6 wells plates according to manufacturer’s protocol. 24 hr post transfection, medium was replaced with fresh medium including G418 drug (1.8 mg/ml) (Hyclone) to select for transfectants. Subsequently, the cells were expanded and subcloned by single cell dilution. Clones were then validated by IFA in 96 wells plate as follow: cells were fixed with 1% paraformaldehyde, 15 min at 37 °C and washed with PBS. The cells were then incubated for 1hr at 37 °C with chicken anti-C-myc antibody (Abcam) or rat anti-pooled CIR antibodies and/or rat preimmune serum (1:10 diluted in PBS 3% BSA). After washing 3 times with PBS, secondary Alexa 594 goat anti-chicken (ImmunoJackson) or Alexa 488 goat anti-rat (Biolegend) (1:600 and 1:500 dilution respectively in PBS 3% BSA) was incubated for 1hr at 37 °C. Nuclei was stained with Hoechst 33342 (1uM in PBS). The fluorescence signal was quantified using the fluorescence plate reader, Infinite M200 PRO multimode reader (TECAN).

### CHO cells Erythrocyte Binding assay

The erythrocyte binding assay on CHO cells was carried out as previously described^46^,^47^. Briefly, the CHO cells seeded in 6-wells plate were incubated with 0.8% hematocrit of washed mouse erythrocytes in complete F12 culture medium supplemented with 10% FBS for 2 hr at 37 °C in 5% CO_2_. The cells were then washed 3 times with sterile PBS and fixed with 1% glutaraldehyde for 10 min at 37 °C to stabilize the rosettes. The cells were washed once again and incubated with Hoechst 33342 (1uM in PBS) for nuclei staining. A rosette was taken into account when five or more mouse erythrocytes bound to a single CHO cell. A total of ~3000 CHO cells were counted using NiKon ECLIPSE 50i microscope with 20X objective. The transfection efficiency was considered as 100% since CHO cells were maintained under G418 drug pressure and validated by IFA. The rosetting frequency (%) was calculated as total no. of rosettes ×100/total no. of counted CHO cells and normalized against the CHO-PeGFP construct (negative control).

For the inhibition assays, CHO cells were incubated in 24 wells plates with 0.05% hematocrit of mouse erythrocytes and antibodies at 1:10 dilution and binding assay was subsequently performed as described above. Inhibition of rosetting was determined as follow: Inhibition rosetting (%) = (Control-(CHO-PCHAS_040110 with antibodies)/control) x100, where control is CHO-PCHAS_040110 cells without antibodies (040110-Ab).

The mouse erythrocytes enzymatic treatment assay was carried out as previously described^22^. Briefly, chymotrypsin (Sigma-Aldrich) and trypsin (Sigma-Aldrich) were used at both 1 and 2 mg/ml and neuraminidase (Roche Diagnostics) at 25 and 50 mU for the treatment of mouse erythrocyte. All treated mouse erythrocytes were washed thrice with F12 medium prior CHO binding assay. Human RBC was washed thoroughly thrice with RPMI prior used for binding.

### Mouse Erythrocyte Binding assay (EBA)

Mouse EBA were carried out as previously described^22^. In brief, about 2.5 μg of proteins purified under non-denaturing condition and dialyzed against PBS were added to 100 μl of packed mouse erythrocytes with 50 μl of FBS, top up with incomplete F12 medium to a total of 600 μl. Sample were incubated for 2 hr at 37 °C with gentle rotating before 600 μl of DBP (dibutylpathalate) oil was overlayed to separate the supernatant (unbound) proteins. After centrifugation, the supernatant and oil layer were removed, bound proteins were then eluted with 0.5 M NaCl. After 10 min incubation at RT and centrifugation, 15 μL of the EBA Eluate was analysed by immunoblot using either mouse monoclonal (Clontech) at 1:10,000 dilution or polyclonal anti-His antibody (Qiagen) at 1:1000. It is important to note that, to maximize our chances of detection, more proteins from the EBA eluate than the purified proteins were loaded on the SDS PAGE gel. It is therefore not a quantitative comparison.

### ELISA based erythrocyte-binding assay

PCHAS_000730, _140090, _110020, and _000950 were purified by gel elution as described above and re-solubilized in PBS. PCHAS_000100, PCHAS_040110 and *P. falciparum* calmodulin were directly purified under non-denaturing conditions and dialyzed against PBS before use. Blood from naive mice was then collected and leukocytes were removed using Plasmodipur filters (Euro-diagnostica). 2 × 10^6^ mouse red blood cells were added in each well of a V-bottom 96 wells plate (NUNC) and blocked for 30 min on ice with PBS, 3% BSA. Cells were sequentially washed three times with PBS and incubated: i) 1h on ice with 100 μL of twofold serial dilutions of histidine-tagged recombinant proteins, ii) 30 min on ice with 50 μL of mouse anti-histidine or rat anti-Ter119 diluted at 1/250 in PBS, 3% BSA, and iii) 30 min on ice with 50 μL of Alexa fluor 488 anti-mouse or anti-rat IgG (Sigma) diluted at 1/1000 in PBS, 3% BSA. Samples were acquired on a BD LSRII flow cytometer, and data were analysed with FlowJo software (TreeStar).

### Statistical analysis

Statistical analysis was determined by using Graphpad Prism5 software. The non-parametric Mann-Whitney U test and One way ANOVA with a post hoc (Bonferroni) test were used. *P*-value of: *<0.05, **<0.01, ***<0.001 were considered as statistically significant.

### In silico analysis

Genomic and protein sequence of genes were obtained from PlasmoDB (http://plasmodb.org) and GeneDB (http://www.genedb.org). Transmembrane domains were predicted using TMHMM 2.0 and signal sequences were predicted using SignalIPv.30. Alignment of protein sequences was done using ClustalW 2.1.

## Additional Information

**How to cite this article**: Yam, X. Y. *et al.* Characterization of the *Plasmodium* Interspersed Repeats (PIR) proteins of *Plasmodium chabaudi* indicates functional diversity. *Sci. Rep.* **6**, 23449; doi: 10.1038/srep23449 (2016).

## Supplementary Material

Supplementary Information

## References

[b1] Scherf A., Lopez-Rubio J. J. & Riviere L. Antigenic variation in Plasmodium falciparum. Annu Rev Microbiol 62, 445–470, 10.1146/annurev.micro.61.080706.093134 (2008). 18785843

[b2] Rowe J. A., Claessens A., Corrigan R. A. & Arman M. Adhesion of Plasmodium falciparum-infected erythrocytes to human cells: molecular mechanisms and therapeutic implications. Expert Rev Mol Med 11, e16, 10.1017/S1462399409001082 (2009). 19467172 PMC2878476

[b3] Smith J. D., Rowe J. A., Higgins M. K. & Lavstsen T. Malaria’s deadly grip: cytoadhesion of Plasmodium falciparum-infected erythrocytes. Cell Microbiol 15, 1976–1983, 10.1111/cmi.12183 (2013). 23957661 PMC3836831

[b4] Cunningham D., Lawton J., Jarra W., Preiser P. & Langhorne J. The pir multigene family of Plasmodium: Antigenic variation and beyond. Molecular and Biochemical Parasitology 170, 65–73 (2010). 20045030 10.1016/j.molbiopara.2009.12.010

[b5] Janssen C., Phillips R., Turner C. & Barrett M. Plasmodium interspersed repeats: the major multigene superfamily of malaria parasites. Nucleic Acids Res 32, 5712–5720 (2004). 15507685 10.1093/nar/gkh907 PMC528792

[b6] Tachibana S.-I. *et al.* Plasmodium cynomolgi genome sequences provide insight into Plasmodium vivax and the monkey malaria clade. Nat Genet 44, 1051–1055, doi: http://www.nature.com/ng/journal/v44/n9/abs/ng.2375.html#supplementary-information (2012). 22863735 10.1038/ng.2375 PMC3759362

[b7] Bernabeu M. *et al.* Functional analysis of Plasmodium vivax VIR proteins reveals different subcellular localizations and cytoadherence to the ICAM-1 endothelial receptor. Cellular Microbiology 14, 386–400, 10.1111/j.1462-5822.2011.01726.x (2012). 22103402

[b8] Carvalho B. O. *et al.* On the cytoadhesion of Plasmodium vivax-infected erythrocytes. The Journal of infectious diseases 202, 638–647, 10.1086/654815 (2010). 20617923

[b9] Goel S. *et al.* RIFINs are adhesins implicated in severe Plasmodium falciparum malaria. Nat Med 21, 314–317, 10.1038/nm.3812 (2015). 25751816

[b10] Niang M. *et al.* STEVOR is a Plasmodium falciparum erythrocyte binding protein that mediates merozoite invasion and rosetting. Cell Host Microbe 16, 81–93, 10.1016/j.chom.2014.06.004 (2014). 25011110 PMC4382205

[b11] Niang M., Yam X. Y. & Preiser P. R. The *Plasmodium falciparum* STEVOR Multigene Family Mediates Antigenic Variation of the Infected Erythrocyte. PLoS Pathog 5, e1000307 (2009). 19229319 10.1371/journal.ppat.1000307 PMC2637975

[b12] Gilks C., Walliker D. & Newbold C. Relationships between sequestration, antigenic variation and chronic parasitism in Plasmodium chabaudi chabaudi - a rodent malaria model. Parasite Immunol 12, 45–64 (1990). 2314922 10.1111/j.1365-3024.1990.tb00935.x

[b13] McLean S. A., Pearson C. D. & Phillips R. S. Antigenic variation in Plasmodium chabaudi: analysis of parent and variant populations by cloning. Parasite Immunol 8, 415–424 (1986). 3774374 10.1111/j.1365-3024.1986.tb00858.x

[b14] Brannan L. R., McLean S. A. & Phillips R. S. Antigenic variants of Plasmodium chabaudi chabaudi AS and the effects of mosquito transmission. Parasite Immunol 15, 135–141 (1993). 8100357 10.1111/j.1365-3024.1993.tb00593.x

[b15] McLean S., Pearson C. & Phillips R. Plasmodium chabaudi - antigenic variation during recrudescent parasitemias in mice. Exp Parasitol 54, 296–302 (1982). 7151940 10.1016/0014-4894(82)90038-8

[b16] Brugat T. *et al.* Sequestration and histopathology in Plasmodium chabaudi malaria are influenced by the immune response in an organ-specific manner. Cellular Microbiology 16, 687–700, 10.1111/cmi.12212 (2014). 24003897 PMC4234010

[b17] Ebbinghaus P. & Krucken J. Characterization and tissue-specific expression patterns of the Plasmodium chabaudi cir multigene family. Malaria Journal 10, 272, 10.1186/1475-2875-10-272 (2011). 21929749 PMC3189184

[b18] Mota M. M., Jarra W., Hirst E., Patnaik P. K. & Holder A. A. Plasmodium chabaudi-Infected Erythrocytes Adhere to CD36 and Bind to Microvascular Endothelial Cells in an Organ-Specific Way. Infect. Immun. 68, 4135–4144 (2000). 10858230 10.1128/iai.68.7.4135-4144.2000 PMC101711

[b19] Mackinnon M., Walker P. & Rowe J. Plasmodium chabaudi: rosetting in a rodent malaria model. Exp Parasitol 101, 121–128 (2002). 12427466 10.1016/s0014-4894(02)00103-0

[b20] Lawton J. *et al.* Characterization and gene expression analysis of the cir multi-gene family of Plasmodium chabaudi chabaudi (AS). BMC Genomics 13, 125, 10.1186/1471-2164-13-125 (2012). 22458863 PMC3384456

[b21] Otto T. D. *et al.* A comprehensive evaluation of rodent malaria parasite genomes and gene expression. BMC Biology 12, 86, 10.1186/s12915-014-0086-0 (2014). 25359557 PMC4242472

[b22] Grüber A. *et al.* Structural Characterization of the Erythrocyte Binding Domain of the Reticulocyte Binding Protein Homologue Family of Plasmodium yoelii. Infection and Immunity 79, 2880–2888, 10.1128/iai.01326-10 (2011). 21482683 PMC3191949

[b23] Cunningham D. A. *et al.* Host immunity modulates transcriptional changes in a multigene family (yir) of rodent malaria. Molecular Microbiology 58, 636–647 (2005). 16238615 10.1111/j.1365-2958.2005.04840.x

[b24] Fonager J. *et al.* Reduced CD36-dependent tissue sequestration of Plasmodium-infected erythrocytes is detrimental to malaria parasite growth *in vivo*. J Exp Med 209, 93–107, 10.1084/jem.20110762 (2012). 22184632 PMC3260870

[b25] Siau A. *et al.* Identification of a new export signal in Plasmodium yoelii: identification of a new exportome. Cellular Microbiology 16, 673–686, 10.1111/cmi.12293 (2014). 24636637

[b26] Rug M., Wickham M. E., Foley M., Cowman A. F. & Tilley L. Correct Promoter Control Is Needed for Trafficking of the Ring-Infected Erythrocyte Surface Antigen to the Host Cytosol in Transfected Malaria Parasites. Infect. Immun. 72, 6095–6105 (2004). 15385514 10.1128/IAI.72.10.6095-6105.2004 PMC517558

[b27] Ingmundson A., Nahar C., Brinkmann V., Lehmann M. J. & Matuschewski K. The exported Plasmodium berghei protein IBIS1 delineates membranous structures in infected red blood cells. Molecular Microbiology 83, 1229–1243, 10.1111/j.1365-2958.2012.08004.x (2012). 22329949 PMC3502748

[b28] Przyborski J. M. The Maurer’s clefts of Plasmodium falciparum: parasite-induced islands within an intracellular ocean. 24, 285–288 (2008). 10.1016/j.pt.2008.04.002 18514031

[b29] Yam X. Y., Mbengue A. & Braun-Breton C. In Malaria Parasites (ed. Omolade Okwa ) (InTech, 2012).

[b30] Petter M. *et al.* Variant proteins of the Plasmodium falciparum RIFIN family show distinct subcellular localization and developmental expression patterns. Mol Biochem Parasitol 156, 51–61, 10.1016/j.molbiopara.2007.07.011 (2007). 17719658

[b31] McLean S. A., MacDougall L. M. & Phillips R. S. Early appearance of variant parasites in Plasmodium chabaudi infections. Parasite immunology 12, 97–103 (1990). 2320383 10.1111/j.1365-3024.1990.tb00939.x

[b32] Cunningham D. *et al.* Rapid Changes in Transcription Profiles of the *Plasmodium yoelii yir* Multigene Family in Clonal Populations: Lack of Epigenetic Memory? PLoS ONE 4, e4285 (2009). 19173007 10.1371/journal.pone.0004285 PMC2628738

[b33] Fernandez-Becerra C. *et al.* Variant proteins of Plasmodium vivax are not clonally expressed in natural infections. Mol Microbiol 58, 648–658 (2005). 16238616 10.1111/j.1365-2958.2005.04850.x

[b34] Joergensen L. *et al.* Surface Co-Expression of Two Different PfEMP1 Antigens on Single Plasmodium falciparum-Infected Erythrocytes Facilitates Binding to ICAM1 and PECAM1. PLoS Pathog 6, e1001083, 10.1371/journal.ppat.1001083 (2010). 20824088 PMC2932717

[b35] Kyes S. A., Rowe J. A., Kriek N. & Newbold C. I. Rifins: a second family of clonally variant proteins expressed on the surface of red cells infected with Plasmodium falciparum. Proc Natl Acad Sci USA 96, 9333–9338 (1999). 10430943 10.1073/pnas.96.16.9333 PMC17783

[b36] Oliveira T., Fernandez-Becerra C., Jimenez M. C., Del Portillo H. & Soares I. Evaluation of the acquired immune responses to Plasmodium vivax VIR variant antigens in individuals living in malaria-endemic areas of Brazil. Malaria Journal 5, 83 (2006). 17026752 10.1186/1475-2875-5-83 PMC1626480

[b37] Abdel-Latif M. S., Cabrera G., Kohler C., Kremsner P. G. & Luty A. J. Antibodies to rifin: a component of naturally acquired responses to Plasmodium falciparum variant surface antigens on infected erythrocytes. Am J Trop Med Hyg 71, 179–186 (2004). 15306707

[b38] Fernandez V., Hommel M., Chen Q., Hagblom P. & Wahlgren M. Small, clonally variant antigens expressed on the surface of the Plasmodium falciparum-infected erythrocyte are encoded by the rif gene family and are the target of human immune responses. J Exp Med 190, 1393–1404 (1999). 10562315 10.1084/jem.190.10.1393 PMC2195703

[b39] Lee W. C. *et al.* Glycophorin C (CD236R) mediates vivax malaria parasite rosetting to normocytes. Blood 123, e100–109, 10.1182/blood-2013-12-541698 (2014). 24652986 PMC4007619

[b40] Preiser P. R., Jarra W., Capiod T. & Snounou G. A rhoptry-protein-associated mechanism of clonal phenotypic variation in rodent malaria. Nature 398, 618–622, 10.1038/19309 (1999). 10217144

[b41] Hensmann M. *et al.* Disulfide bonds in merozoite surface protein 1 of the malaria parasite impede efficient antigen processing and affect the *in vivo* antibody response. Eur J Immunol 34, 639–648, 10.1002/eji.200324514 (2004). 14991593

[b42] McKean P. G., O’Dea K. & Brown K. N. A single amino acid determines the specificity of a monoclonal antibody which inhibits *Plasmodium chabaudi* AS *in vivo*. Mol Biochem Parasitol 62, 211–221 (1993). 7511215 10.1016/0166-6851(93)90110-j

[b43] Huang X. *et al.* The Role of Serine-Type Serine Repeat Antigen in Plasmodium yoelii Blood Stage Development. PLoS ONE 8, e60723, 10.1371/journal.pone.0060723 (2013). 23634205 PMC3636278

[b44] Janse C. J. *et al.* High efficiency transfection of Plasmodium berghei facilitates novel selection procedures. Molecular and Biochemical Parasitology 145, 60–70, 10.1016/j.molbiopara.2005.09.007 (2006). 16242190

[b45] Spence P. J. *et al.* Transformation of the rodent malaria parasite *Plasmodium chabaudi*. Nat Protoc 6, 553–561, 10.1038/nprot.2011.313 (2011). 21455190 PMC3968397

[b46] Chitnis C. & Miller L. Identification of the erythrocyte binding domains of *Plasmodium* vivax and Plasmodium knowlesi proteins involved in erythrocyte invasion. J Exp Med 180, 497–506 (1994). 8046329 10.1084/jem.180.2.497 PMC2191600

[b47] Gao X. *et al.* Antibodies targeting the PfRH1 binding domain inhibit invasion of *Plasmodium* falciparum merozoites. PLoS pathogens 4, e1000104, 10.1371/journal.ppat.1000104 (2008). 18617995 PMC2438614

