# Supplementary material for: Characterization of the Plasmodium Interspersed Repeats (PIR) proteins of Plasmodium chabaudi indicates functional diversity
Source: Sci Rep. 2016 Mar 21;6:23449. doi: 10.1038/srep23449 (PMC4800443; doi:10.1038/srep23449)
Supplement: Supplementary Information [file srep23449-s1.pdf]

Supplementary Information

Characterization of the *Plasmodium* Interspersed Repeats (PIR) proteins of *Plasmodium chabaudi* indicates functional diversity

Xue Yan Yam[1+], Thibaut Brugat[2+], Anthony Siau[1], Jennifer Lawton[2,], Daniel. S.Wong[1], Abdirahman Farah[1,3], Jing Shun Twang[1], Xiaohong Gao[1], Jean Langhorne[2*], Peter R. Preiser[1*]

[1]: School of Biological Sciences, Nanyang Technological University, 637551, Singapore

[2]: Francis Crick Institute, Mill Hill Laboratory, London, UK

[3]: Karolinska Institutet, Stockholm, Sweden

Supplementary Data

A

Gene I.D.	CIR subfamily classification	Region of protein expressed (a.a)	Theoretical M.W.	Theoretical p.I.
PCHAS_000730	S	24-238	26	5.77
PCHAS_140090	S	51-235	23	5.3
PCHAS_110020	S	23-233	26	7.6
PCHAS_000100	S	1-232	28	5.46
PCHAS_000950	L	54-306	30	6.03
PCHAS_040110	L	1-269	32	6.21

B

	Motifs	Frequency in subfamilies		Peptides synthesised
		L	S	
CIR-L	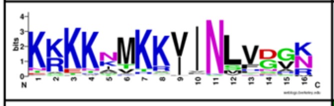	93.44	0.00	KNMKKVINLAYGK
CIR-S	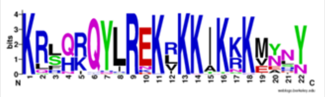	0.00	80.72	QYLREKRKKAKRKVYNY

C

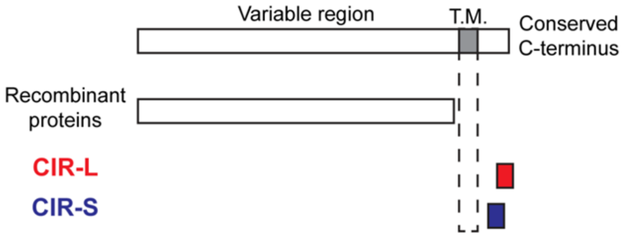

Fig S1: Production of recombinant CIR proteins and subfamily-specific peptides

A) Table summarizing the characteristics of the His-tagged recombinant proteins expressed for this study. The gene I.D., CIR-subfamily, region of the protein expressed and the theoretical molecular weight (M.W.) and isoelectric point (p.I.) of the recombinant proteins are indicated. **B)** Conserved amino-acid motifs within the CIR repertoire were identified by MEME analysis by

Lawton et al [19] and shown here as Weblogo images. Blue: hydrophobic residues; Green: polar, non-charged residues; Purple: acidic residues; and Red: positively charged residues. The percentage of CIRs containing each motif within subfamilies L and S and the sequence of the peptides synthesized for antibody production are shown. C**)** Position of the regions selected for protein and peptide production on a typical CIR protein. TM: transmembrane domain (shown in grey).

```
PCHAS_000730_S7_      ----------------------------MLAFIEFDNILTEYCTEEEGGGKKECFSYEVL 32
PCHAS_110020_S7_      ----------------------------MKQLFHYNTGSYPYCINGEGG-TQECFSYEVI 31
PCHAS_140090_S7_      ----------------------------MIKITN---------------------SAIIA 11
PCHAS_000100_S7_      MRDPSYKIEDVYNDIFKISNYFDEDENDGTKTKVTNKAIHNYCHYDDRQEKDKCNDYYEM 60
PCHAS_000950_L2_      ----------------------------------------------------MGAMFKLL 8
PCHAS_040110_L4_      -------------MSKELCQGINFADKNVVFDQKSESYKFNDDIFKVYCPNNNCDSNGLK 47

PCHAS_000730_S7_      ISSIFIKLLKIFMNLDD--LENDKHAQYAILWFCHKLNKNSQNGINNLNDVYNKYIKGNK 90
PCHAS_110020_S7_      VSSLFIKMVNHFMKGDD--LKNNKLAEYAILWLCYKLNKKKENEISNLNDFHNKYIKGNE 89
PCHAS_140090_S7_      VLNYFKNIS--VADGDD--LEDDKIAEYAILWLNHKLNQYPEYGVSNLDDFHNTYIKD-- 65
PCHAS_000100_S7_      TSSGVIHLINNLKGKNV--LDYDKLAEYAILWLSYKLKIKENPIIKKLSVFYDSYIERNE 118
PCHAS_000950_L2_      LQQLFVNNEGDLELEN----KNDEYSEYAILWLSNIIRHFNYKHTPHVQDFYTTFIKE-- 62
PCHAS_040110_L4_      IGSAFTALLEYFKNIDNENSEDDKLTQYAILWFNSKLSQKPDIEIERYTMYN-------- 99
                        . .      .   :    . :: ::*****:   :         .

PCHAS_000730_S7_      EFFEKINDAGAYNSYEEFINKKQDMVNVNIKDMSKFYEALQILCKMYTKIDESKSNCTKC 150
PCHAS_110020_S7_      NHIDKINGAGSYNSYKNIINNKKYLNTIDIKEMSKFYVPLKSLCKLYTEYSKKKKNYTTC 149
PCHAS_140090_S7_      --IKETTDIEGYNAYMDFIEKKQDMMNMDIKDMSKFYASLKSLCDMQTKIDANKSNCASY 123
PCHAS_000100_S7_      YYNKNINGDE--LTYKEIIDKKKDLMDMDINEIFKLEAPFNILYYLYHQIYDKNSFCSEY 176
PCHAS_000950_L2_      --------GNWYNDFRDKINKKNDIMKLQINQIYNLYELLNILCKAITKYDENPSNSSEC 114
PCHAS_040110_L4_      ----ILKQYNWFGEDGKSIEKKKDIMGIHYLYLKNLYDFLKGICETITNCN-GHSKTSEC 154
                                      . *::*: :  :.   : ::   :: :     :     .  :

PCHAS_000730_S7_      LDDAQKFADEY-EKINSNS-ITGNDSYSQMLSNLFVDYDNLKNKC--KNSSSLPTIKKPQ 206
PCHAS_110020_S7_      SQDAQDFAKHF-EDLNQDSSITENISYREILSSLSIDYDDFKNECA-KNCSYCNDIPTLS 207
PCHAS_140090_S7_      LEDSQKFVDEY-EKLNENY-DTKGVSYKKILSTLFNDYDRLRKKC--NNFQSFPSMKITQ 179
PCHAS_000100_S7_      SDYANQFVQKF-EDLNNDPNNKEDSSYNKLLSILSDDYNNLQKKC--TNFPSLPVYPR-- 231
PCHAS_000950_L2_      SNFANKWEERSKELVNKETKVFEDEHYCNALLNLKKAYEKFTNGNNTNAFPKINEIEKMN 174
PCHAS_040110_L4_      IESAKKCSGLYRACILHLPWREICNPYCNVLTNLKNDYDKIRENNRDKKLPELTLPEGLY 214
                       : ::.        :           * : *  *   *: : :    .

PCHAS_000730_S7_      NFEQRFE----------------------------------------------------- 213
PCHAS_110020_S7_      EIK--------------------------------------------------------- 210
PCHAS_140090_S7_      SYVQD------------------------------------------------------- 184
PCHAS_000100_S7_      ------------------------------------------------------------
PCHAS_000950_L2_      NCKKLCEKANRSWKVIHVEVKDVVDAKKVSENGESKKEGEEKTKESNPVETPGPVAILSN 234
PCHAS_040110_L4_      DCKTECYKQEEGHKARVAALQRSSDGSEIDTTPG-------------------------- 248

PCHAS_000730_S7_      -----------------VTSLEHHHHHH 224
PCHAS_110020_S7_      ------------------TPLEHHHHHH 220
PCHAS_140090_S7_      ------------------SDLEHHHHHH 194
PCHAS_000100_S7_      ---------------------SHHHHHH 238
PCHAS_000950_L2_      TGDTSQNDAIKVRADESGENLEHHHHHH 262
PCHAS_040110_L4_      ISLLDLSVPPTSINNGNKLPYIHHHHHH 276
                                            ******
```

Fig S2: Alignment of the 6 His-tagged recombinant proteins

Alignment of protein sequences was performed using ClustalW. Colours determine the different amino acids groups. Highlighted box showed the highly conserved peptide motif.

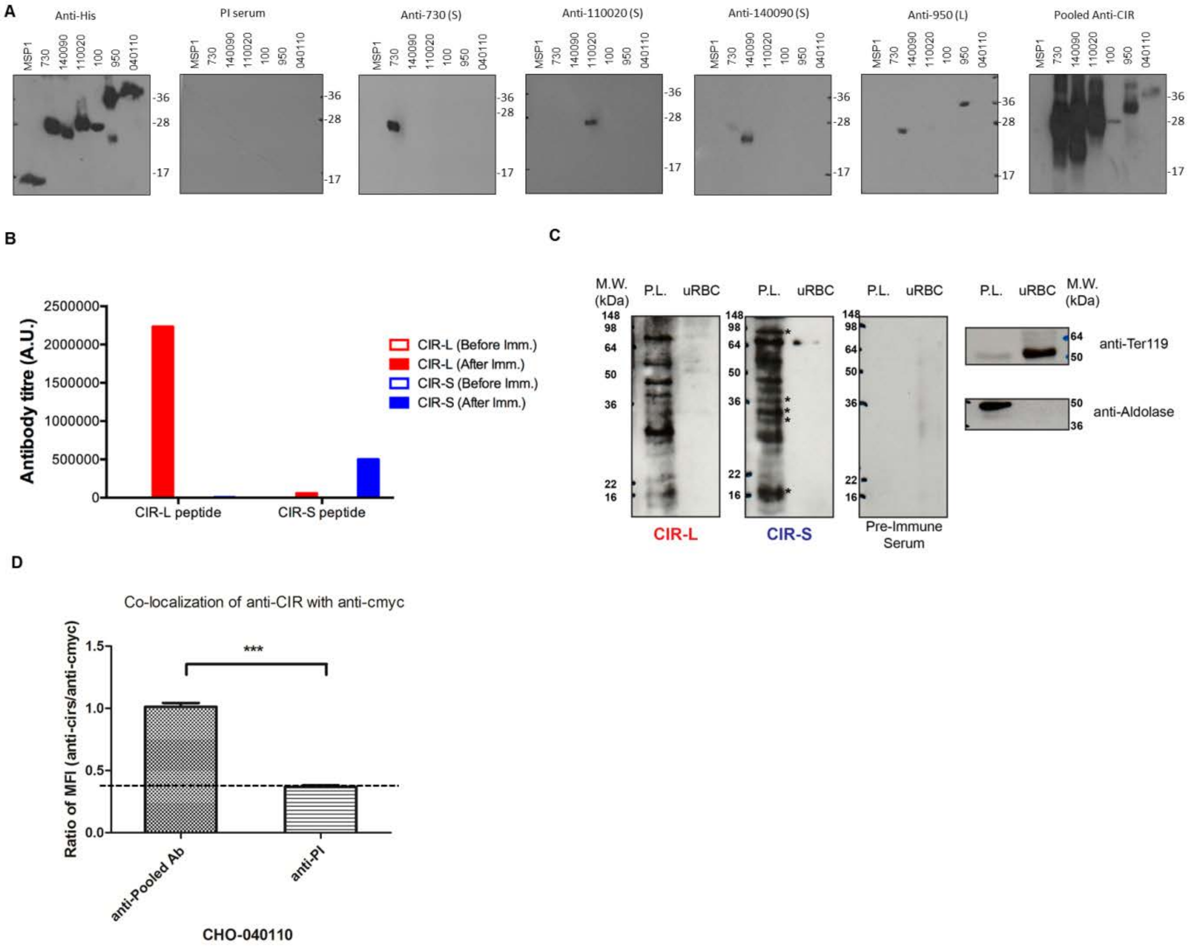

Fig S3: Production of anti-CIR antibodies

A) Western blot experiments were performed to determine the specificity of CIR-specific polyclonal antibodies. The name of the recombinant proteins loaded on the SDS PAGE gel and antibodies used are indicated above, and the molecular weight on the side. Anti-His monoclonal antibody and rat pre-immune (P.I.) serum were used as loading and negative control respectively. **B)** The titres of anti-peptide antibodies in rabbit sera before and after immunization ("Before Imm." and "After Imm." respectively) were determined by ELISA assays. Bars represent antibody titres in arbitrary unit (A.U.). **C)** Western blot experiments were performed to determine the specificity of anti-peptide antibodies in rabbit sera before ("pre-immune serum") and after immunization (CIR-L and CIR-S). Asterisks indicate the differences observed between

the anti-CIR-L and anti-CIR-S antisera. Anti-Ter119 (specific to red blood cells membrane) and anti-Aldolase (soluble protein) antibodies were used as loading controls. P.L.: parasite lysate; uRBC: uninfected red blood cells membranes; M.W.: molecular weight. **D)** IFA was performed on transfected CHO cells expressing PCHAS_040110 proteins. Ratio of Mean fluorescence intensity (MFI) of pooled anti-CIR antibodies/anti-C-myc antibodies was measured using fluorescence plate reader. Bars represent the mean±S.E.M. of three independent experiments. (T-test, ***: $P<0.001$).

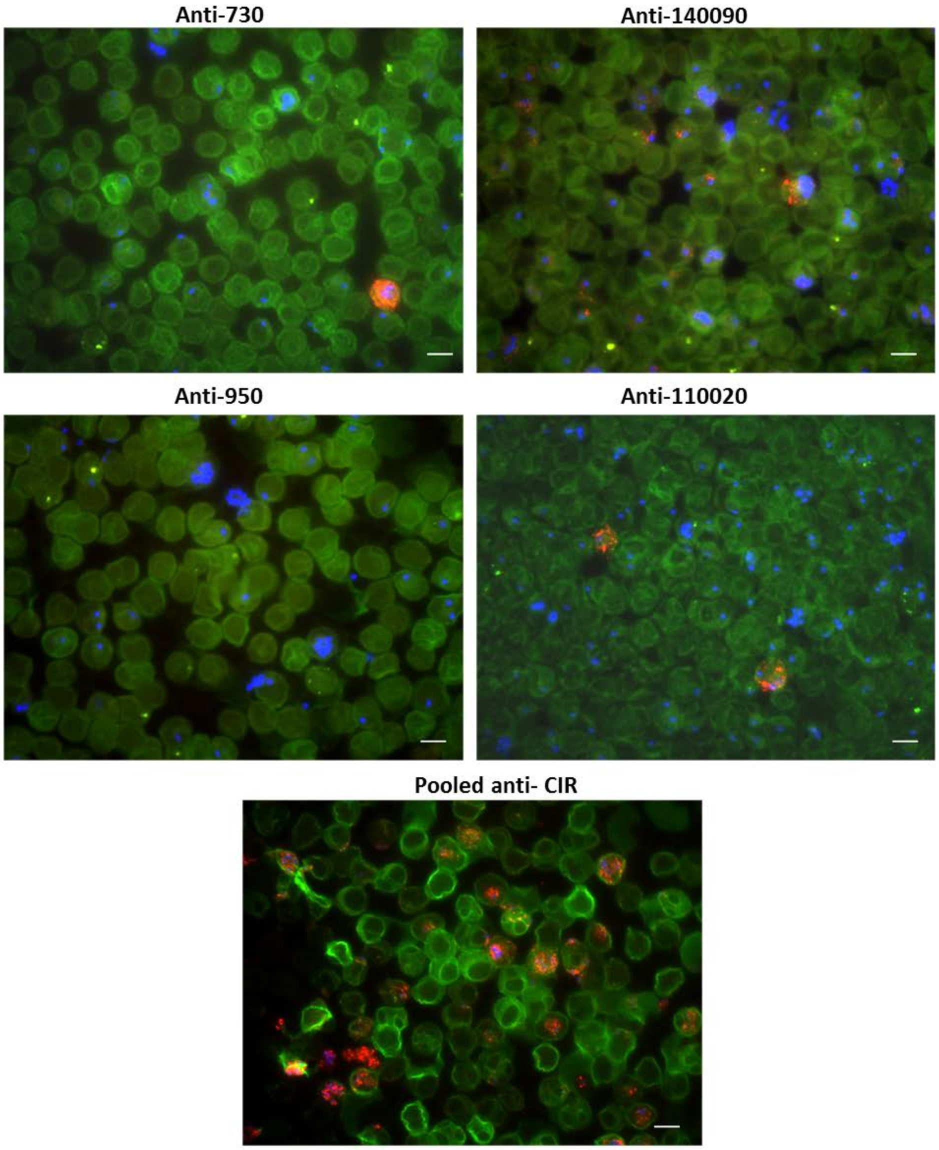

Fig S4: Immunofluorescence experiments using CIR-specific polyclonal antibodies Representative pictures of immunofluorescence assay on acetone/methanol fixed red blood cells (RBC) infected with *P.chabaudi* AS. Parasites nuclei were labeled with Hoechst 33342 (blue) and the mouse erythrocyte membranes with anti-Ter119 antibody (green). CIR proteins were labeled with either a single CIR-specific polyclonal antibodies or a pool of polyclonal anti-CIR antibodies (red). Scale bars represent 5 µm.

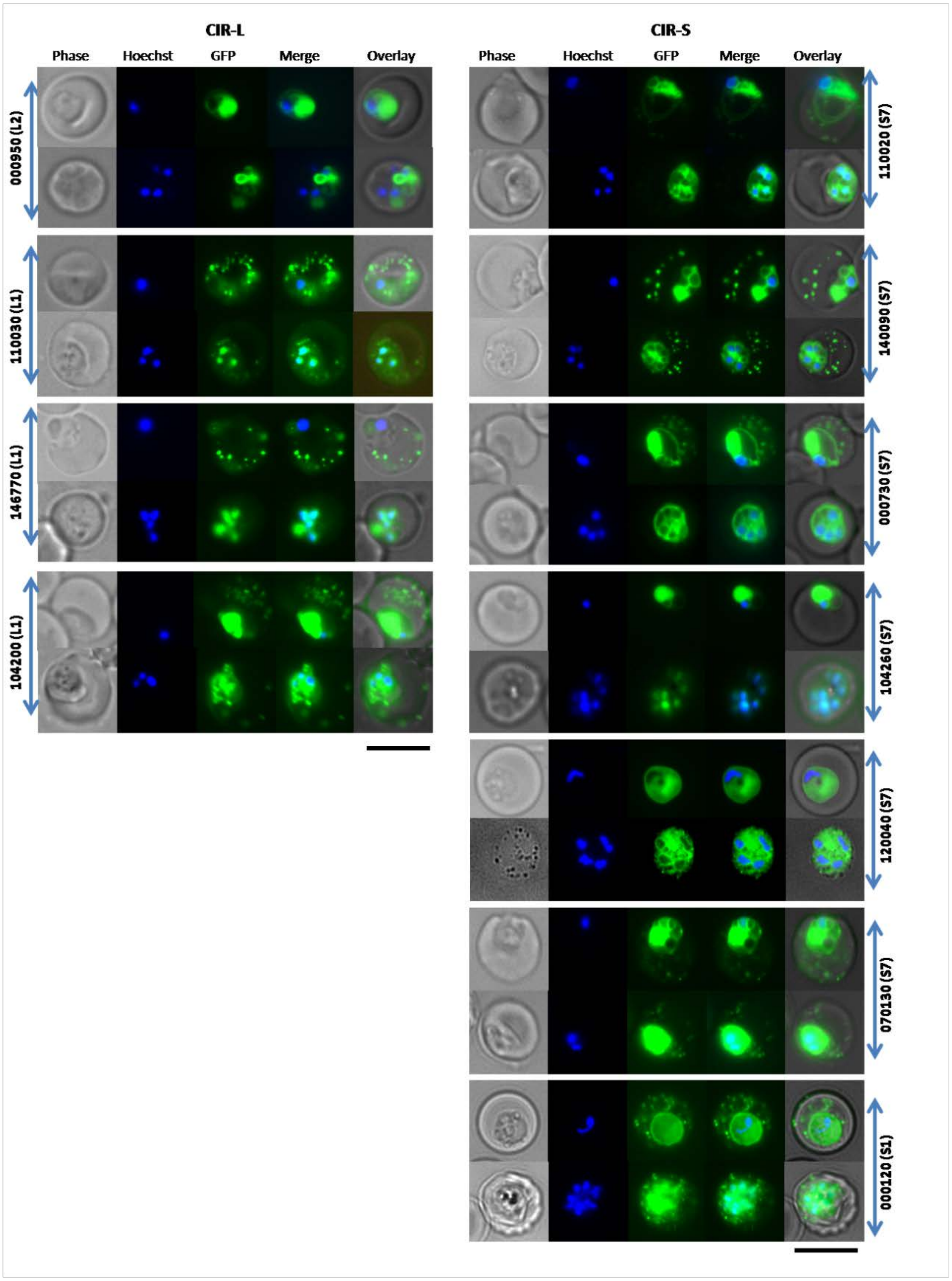

Fig S5: Live imaging of *P.chabaudi* parasites expressing CIRs-GFP tagged

Live cell imaging of transgenic *Plasmodium chabaudi* parasites expressing CIR proteins tagged with GFP (green). Representative pictures of the trophozoite (upper panel) and schizont (lower

panel) stages of each CIR member are shown. The subfamilies and gene accession number of the each CIR member are indicated along the side of both panels. Parasites nuclei were labeled with Hoechst 33342 (blue). The phase contrast image is shown in grey. Scale bars represent 5µm.

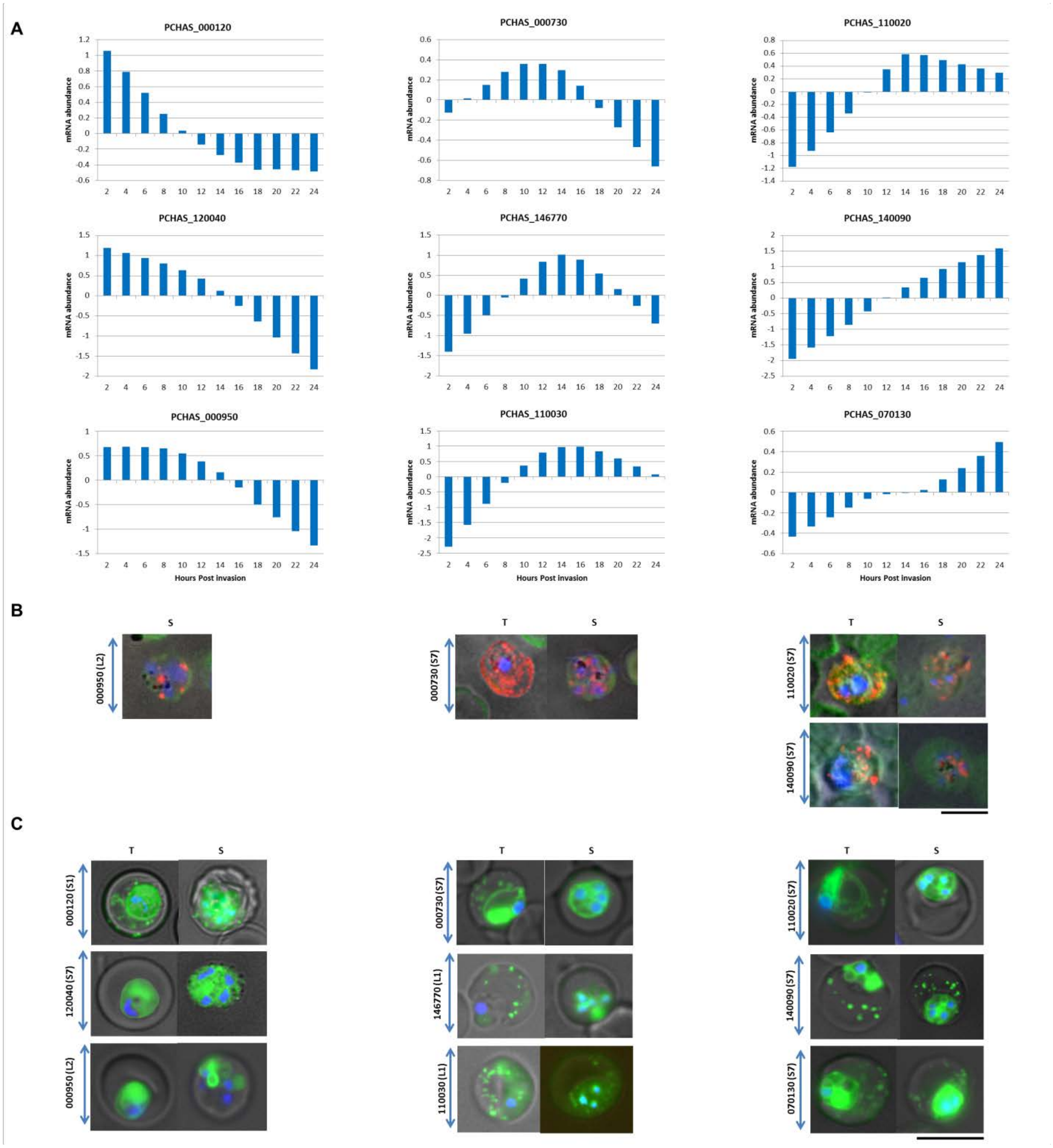

Fig S6: Comparison of *cir* transcriptional profile with CIR localization

A) The transcriptional graph of each *cir* gene is plotted using the raw microarray data from Lawton *et al.*, *BMC genomics* 2012, with mRNA abundance against hours post parasite invasion. First panel represent genes which are early transcribed (Ring/early trophozoites (ET) stage),

second panel are mid to late-trophozoites transcribed and last panel are late parasite stage transcribed. **B)** Representative pictures of immunofluorescence assay on acetone/methanol fixed red blood cells (RBC) infected with *P.chabaudi* AS. Parasites nuclei were labeled with Hoechst 33342 (blue) and the mouse erythrocyte membranes with an anti-Ter119 antibody (green). CIR proteins were labeled with individual polyclonal anti-CIR antibodies (red) in trophozoite (T) or schizont (S). First panel represent CIR member that is transcribed in early parasite stage (Ring/early trophozoites (ET)); second panel represent CIR member transcribed in trophozoite stage and last panel represent CIR members transcribed in the late parasite stage. **C)** Representative pictures of the trophozoite (T) and schizont (S) stages of each GFP-tagged CIR member are shown. Parasites nuclei were labeled with Hoechst 33342 (blue). The subfamilies and gene accession number of the each CIR member are indicated along the side of the pictures. First panel corresponds to the early transcribed genes; second panel to the mid to late trophozoites and last panel to the late parasite transcribed genes respectively to their transcription data as shown in (A). Scale bar represent 5 μm.

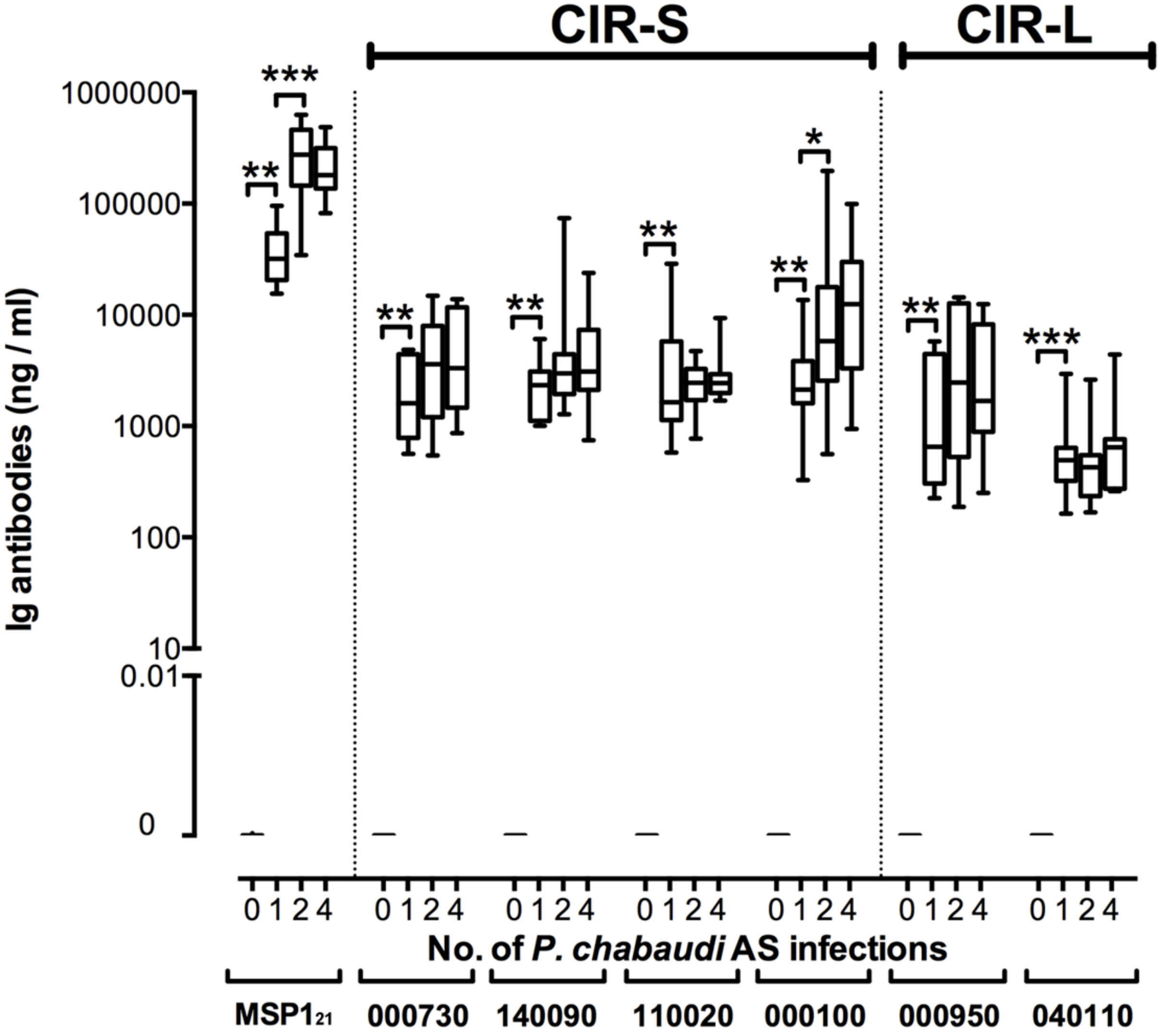

Fig S7: CIR proteins are recognized by the host immune system during infection

ELISA assays were performed to quantify the amount of CIR-specific Ig antibodies present in the plasma of C57BL/6 mice before (0) or after 1, 2 or 4 infections with *P. chabaudi* (n=10 mice per group). The name of the recombinant proteins used is indicated below (see also Fig S1A and S2). $MSP1_{21}$ recombinant protein was used as a positive control. Columns represent the median with interquartile range. Bars represent minimum and maximum values. (Mann-Whitney; *: $P<0.05$; **: $P<0.01$; ***: $P<0.001$)
